# The Reciprocal Relationship between Climate and Environmental Changes and Food Systems and Its Impact on Food/Nutrition Security and Health

**DOI:** 10.3390/nu15132824

**Published:** 2023-06-21

**Authors:** Andrew A. Bremer, Daniel J. Raiten

**Affiliations:** Pediatric Growth and Nutrition Branch, *Eunice Kennedy Shriver* National Institute of Child Health and Human Development, National Institutes of Health, US Department of Health and Human Services, 6710B Rockledge Drive-Rm 2444, Bethesda, MD 20892, USA

**Keywords:** climate/environmental change, food systems, food/nutrition insecurity, nutritional ecology, nutrition and health

## Abstract

Changes in our climate and physical environments are having profound effects on all aspects of human existence, and the ability to develop sustainable and resilient food systems is critical not just to the environment but to all aspects of human health. The Pediatric Growth and Nutrition Branch (PGNB) of the *Eunice Kennedy Shriver* National Institute of Child Health and Human Development of the US National Institutes of Health has adopted a new paradigm to undergird the study of nutrition that recognizes the complex and reciprocal nature of the relationships between nutrition and health outcomes. This conceptual framework, termed the “nutritional ecology,” views humans as complex biological systems interacting with both their internal and external environments. Herein, we focus on: (i) the reciprocal relationship between climate and environmental changes and food systems and their impact on food/nutrition security and health; and (ii) how PGNB is utilizing the “nutritional ecology” framework to support science addressing the interactions among health, nutrition, food systems, climate, and the environment.

## 1. The “Nutritional Ecology”

Our world has become increasingly complex, affecting our ability to support a growing and diverse population at a global scale. Furthermore, this complexity needs to be appreciated and integrated into our efforts to address the challenges presented by a changing global environment. We within the Pediatric Growth and Nutrition Branch (PGNB) of the *Eunice Kennedy Shriver* National Institute of Child Health and Human Development (NICHD)—part of the US National Institutes of Health (NIH)—have adopted a new paradigm to undergird the study of nutrition that recognizes the complex and reciprocal nature of the relationships between nutrition and health outcomes (i.e., nutrition both affects and is affected by an individual’s overall health). This conceptual framework, which we term “nutritional ecology”, views humans as complex biological systems interacting with both their “internal” (i.e., biology, genetics, health, and nutrition) and “external” (i.e., social, behavioral, cultural, and physical) environments [1]. The “nutritional ecology” paradigm also integrates the view of nutritional status as a biological variable that is appreciated both as an input that affects susceptibility to disease and response to treatment as well as an output that reflects the outcome of disease and treatment [1].

**Figure 1:**
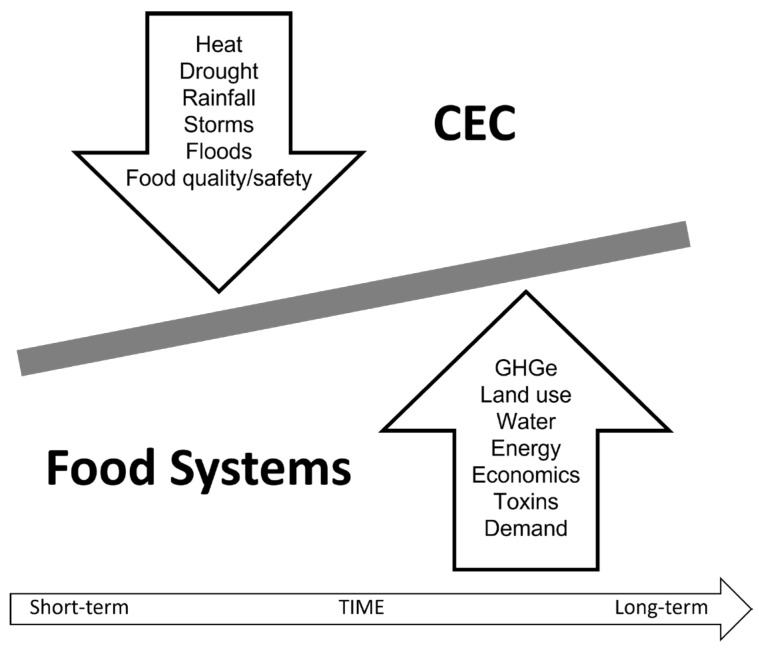
Reciprocal interactions between food systems and climate and environmental change (CEC). Specifically, CEC impacts food systems and food systems impact CEC. GHGe: greenhouse gas emissions.

Herein, we make the case for the value added by using this ecological approach to address the challenges of food/nutrition insecurity in the context of an increasingly complex and changing environment. We take a holistic view of the environment and appreciate that it encompasses: (i) an individual’s home and community; (ii) an individual’s underlying biology, genetics, life stage, and health; (iii) social, economic, and political influences; and (iv) natural land, water, and climate. These elements of the environment are dynamic, and many are constantly in flux. Moreover, the environment impacts and is impacted by food systems, which we define as all processes and infrastructure involved in feeding a population (i.e., growing, harvesting, processing, packaging, transporting, marketing, consuming, and disposing of food and food-related items). For this particular manuscript, we focus on the reciprocal relationship between climate and environmental changes (CECs) and food systems (Figure 1) and, ultimately, the impact of these relationships on nutrition and health.

## 2. Context

**Figure 2:**
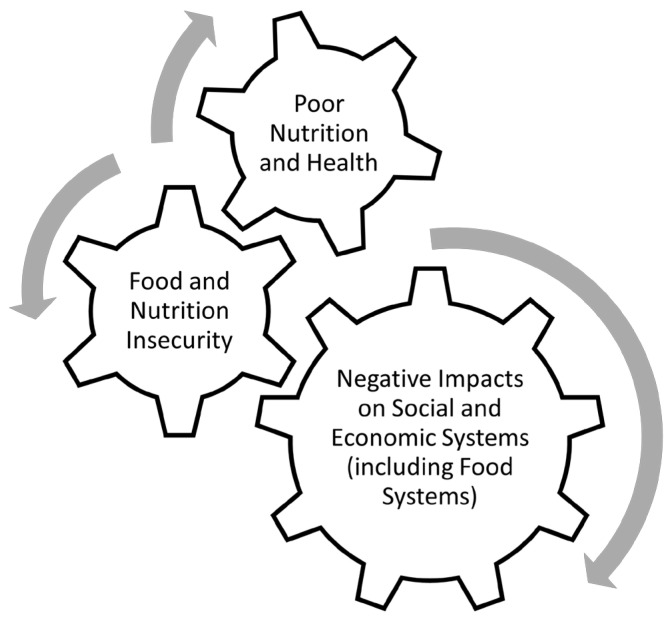
Vicious cycle of food/nutrition insecurity, poor health outcomes, and negative impacts on social and economic systems.

Food and nutrition insecurity are the result of a multiplicity of perturbations in structural systems that typically affect the quantity and quality of food in each setting. Importantly, these perturbations can range from: (i) pre-harvest agricultural practices; to (ii) food production; to (iii) post-harvest storage and processing; to (iv) distribution, marketing, and retail. In most contexts, food/nutrition insecurity reflects limitations (accessibility and availability) of both food supply and quality. In the context of the “nutritional ecology”, these factors reflect components of the “external” environment. Importantly, although food/nutrition insecurity affects an individual’s nutritional status, the terms are not interchangeable, and food security should not be conflated with nutrition security. That said, the result of these insecurities can often lead to a vicious cycle of hunger, poor nutrition, poor health, decreased functional capacity of affected populations, and negative impacts on social and economic systems (Figure 2). For example, both food insecurity (lack of overall food) and nutrition insecurity (lack of nutritious food) can negatively impact an individual’s overall health. This can then negatively impact social and economic systems if the individual is unable to work or requires additional resources due to their poor health. And in the event that the individual works in a food supply chain, the underlying food and nutrition insecurity can potentially negatively impact food systems.

Temporally, challenges impacting food/nutrition insecurity can be chronic or acute. In the former case (chronic food/nutrition insecurity), the systems responsible for addressing context-specific and equitable solutions to the access and availability of a safe, diverse, and high-quality food supply are often compromised due to economics, production, and resource limitations (including those due to long-term impacts of a changing physical environment) requiring social/political policy and program interventions. In the latter scenario (acute food/nutrition insecurity), numerous types of crises precipitate such limitations, including social/economic turmoil, geopolitical conflicts, pandemic disease, or increasingly, climate change–related disasters [2].

**Figure 3:**
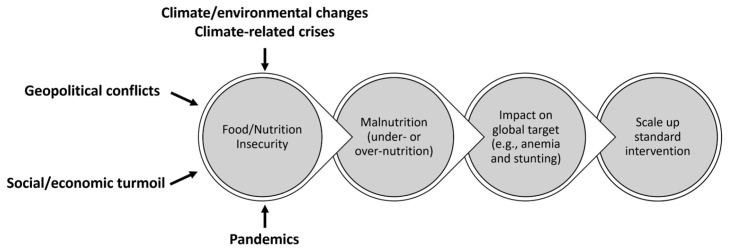
Linear logic model in the response to crises affecting food and nutrition.

For the global food, nutrition, and public health communities, these challenges typically engender a linear logic in the response (Figure 3). This linear logic is relevant to addressing the predominant issue (i.e., food supply) and understandably focuses on programs and policies. However, food safety is also an important issue. Moreover, we live in a complex global health context that includes pre-existing malnutrition intertwined with pandemics, food-borne diseases, and the growing epidemics of non-communicable diseases. This global health context also includes the expanding prevalence of the “multiple burdens” of malnutrition (see below). Thus, the focus of this linear logic primarily on food supply (i.e., “too much or too little”) is limited with respect to addressing specific diet, nutrition, and public health issues because it does not address the biological (“internal” environment) realities of health and disease.

A recent example of this linear logic has been the global response to the COVID-19 pandemic. While there was no doubt that a focus on food/nutrition insecurity was warranted given the profound impact the pandemic had on all aspects of the food system [3], the primary focus of the response on food supply did not allow for: (i) consideration of the actual role of the biology of nutrition on susceptibility or response to treatment for SARS-CoV-2 infection; or (ii) the impact of SARS-CoV-2 infection on the biology of nutrition. Similarly, the global nutrition and health community has understandably concentrated a tremendous amount of energy and resources on addressing global targets such as anemia and stunting, particularly as the pandemic affected food systems. However, these conditions are multidimensional and not limited in etiology to simply food supply. Thus, our response to the impact of the COVID-19 pandemic per se and its indirect effects on priority health outcomes demands an ecological approach to address both short-term health outcomes (including potential unintended consequences) as well as the development of resilient responses that might be applied to similar challenges in the future.

To summarize, in the continuum of evidence-informed programs, guidance, and interventions, such challenges require a resilient approach to the development of new evidence to inform effective responses that are safe, equitable, and efficacious and to avoid unintended consequences. The “nutritional ecology” paradigm provides such a construct and is a value-added approach to the ongoing efforts to address food/nutrition insecurity and their health consequences.

## 3. Global Challenges

Context matters, and the traditional “one-size-fits-all” approach to food/nutrition insecurity by increasing food/nutrient supply does not suffice to either: (i) address the constellation of issues involved at the intersection of diet, nutrition, and health; or (ii) account for potential unintended consequences. Regarding the latter, an intervention may ameliorate one problem while potentially exacerbating others. For example, interventions such as the “Green Revolution” and efforts to increase staple crop production to reduce hunger are obviously laudable. However, they can potentially exacerbate: (i) the reliance on mono-culture agriculture and lack of dietary diversity, leading to the hidden hunger of micronutrient malnutrition and its impact on sustainable food systems [4]; (ii) the balance that will be required to sustainably increase the use of animal-sourced foods to address micronutrient malnutrition, particularly in low-resource settings, while avoiding its potential impact on the climate and physical environment [5]; (iii) safety concerns regarding iron supplementation in the context of malaria [see example below]; and (iv) the multiple burdens of malnutrition and the increased risk of non-communicable diseases such as obesity and diabetes [6].

The challenges associated with adopting a “one-size-fits-all” approach to food/nutrition insecurity are exemplified by the conundrum regarding the safety and efficacy of iron supplementation in populations with a high prevalence of iron deficiency living in settings with endemic infection, most prominently malaria [7]. Specifically, despite well-intentioned interventions, reports indicate potential increased adverse risks when iron is provided in areas with high infection burdens [8]; moreover, the selection and interpretation of biomarkers for assessing iron status are compromised by the inflammatory process [9]. Superimposed on these issues is the impact of climate on this scenario. Specifically, as the climate changes, vector-borne diseases like malaria are projected to increase [10]. At the same time, the changing climate and greenhouse gas emissions negatively impact the nutritional quality of staple crops [11]. Moreover, the available land for growing crops is decreasing—thereby further challenging an already stressed food system in malaria-endemic areas [12]. As a consequence of all these factors, our ability to make context-specific, safe, equitable, and efficacious intervention choices is compromised, again challenging the “one-size-fits-all” approach to public health interventions.

## 4. Climate Change, Food Systems, Food, and Nutrition

**Figure 4:**
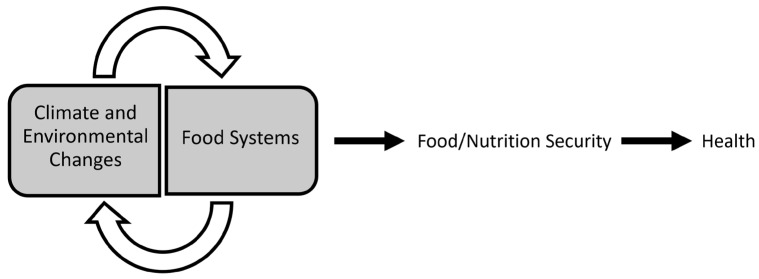
The reciprocal relationship between climate and environmental changes and food systems and its impact on food/nutrition security and health.

As recently noted, changes in our global climate and physical environments are having profound effects on all aspects of human existence [13]. Moreover, the United Nations (UN) views climate change as the defining issue of our time, and the UN’s Secretary-General has called the climate crisis a “code red” for humanity [14]. An emerging area of science that is relevant to this crisis and that is receiving more attention is the intersection of CEC and food systems [13] and, as such, the impact of the reciprocal relationships among CECs, food systems, food/nutrition security, nutrition, and health (Figure 4). Moreover, these relationships can be direct and indirect. For example, CEC can directly impact human health due to the negative sequelae of heat stress. Rising CO_2_ levels in the environment can also indirectly impact human health by modifying the nutritional content of staple crops [15].

In terms of our ability to understand the bilateral relationships between food systems and health, our core operating premise is that nutrition is the glue that ties the food system to health. As mentioned above, it is important to recognize that food/nutrition insecurity are typically issues of supply resulting from the breakdown of structural systems and that food/nutrition insecurity should not be conflated with the biology of nutrition [10]. Specifically, the “supply” issue could potentially be an issue of overall quantity for a population or an issue of proper distribution to those segments of the population in need. Moreover, given the varying degrees of nutritional value of foodstuffs, food security, while related, is not the same as limitations in the quality of food (i.e., nutrition security). Moreover, although food certainly contributes to nutritional status, food is not the same as nutrition, and addressing the former will neither answer questions nor ameliorate the complexities or perturbations of the latter.

For clarity, we define food as sources of nutrients and bioactive substances that are dependent on land/marine food systems or that are commercially processed. Alternatively, we define nutrition as the sum of the processes involved in taking in and utilizing food substances by which growth, repair, maintenance, and reproductive activities of the body or any of its parts are accomplished [1]. The “processes of nutrition” include: (i) ingestion; (ii) digestion; (iii) absorption; (iv) transport; (v) metabolism; and (vi) elimination. Moreover, a reciprocal relationship exists between each of these processes and nutritional status (see below), such that each affects and is affected by the other and may not be ameliorated by simply addressing food/nutrient supply [1]. Finally, we define nutritional status as the relative adequacy of nutrients to perform the various functions of life in an individual; specifically, nutritional status: (i) is achieved as a result of the interaction of food and nutrients with the processes of nutrition; (ii) can affect and/or be affected by these processes, which must be considered in understanding individual nutritional needs; and (iii) varies among individuals and can affect responses to medical and dietary treatments [1].

Many consequences of CEC threaten food systems and food/nutrition security and, thus, expose vulnerable populations to multiple forms of malnutrition, including: (i) undernutrition (underweight, stunting, and wasting); (ii) micronutrient deficiencies; and (iii) overweight and obesity [16]. Importantly, these forms of malnutrition exist in all nations and can coexist within countries, communities, households, and individuals. From a clinical perspective, this is problematic in that malnutrition can adversely affect an individual’s health in myriad ways (including negatively impacting physical and cognitive development, impairing the body’s immune and cellular systems, and increasing the risk of developing communicable and non-communicable diseases) [17]. Moreover, on a global scale, unhealthy diets are major risk factors for diet-related non-communicable diseases (e.g., diabetes, cancer, and cardiovascular disease), and malnutrition in all its forms is the leading cause of morbidity and mortality worldwide [18].

## 5. Moving Ahead

PGNB’s approach to supporting research focusing on the intersection of CEC, food systems, diet, nutrition, and health in NICHD’s target populations is not only in line with the “nutritional ecology” paradigm, but it also aligns with the Biden–Harris Administration’s National Strategy on Hunger, Nutrition, and Health (also referred to as the “National Strategy”) [19,20]. Specifically, our approach addresses Pillar 5 of the National Strategy: enhancing nutrition and food security research by improving nutrition metrics, data collection, and research to inform nutrition and food security policy, particularly on issues of equity, access, and disparities [19,20]. To be even more granular, PGNB’s efforts in this space are directly responsive to a sub-bullet in Pillar 5 of the National Strategy calling for research addressing the intersection of climate change, food security, and nutrition and the United States Department of Health and Human Service’s leverage of the NIH Climate Change and Health (CCH) Initiative to research the effects of climate change on food quality and nutrition security on the health of populations [19]—particularly since NICHD has representation on the Executive and Steering Committees of the NIH CCH Initiative [21].

Furthermore, with specific regard to the challenges presented by the intersection of CEC and food systems and its impact on food/nutrition security and health, the United States Government as a whole has initiated a series of relevant activities that reflect the continuum of experience and expertise across multiple agencies [22,23]. Specifically, PGNB’s efforts in this space—as exemplified by its leadership in the Climate, Health, Agriculture, and Nutrition in a Global Ecology (CHANGE) subgroup of the United States’ Government’s Global Nutrition Coordination Plan 2.0 [22], the Climate/Environmental Change, Health, Agriculture, and Improving Nutrition (CHAIN) Research Interest Section (RIS) of the American Society for Nutrition, and a newly launched initiative called Agriculture and Diet: Value Added for Nutrition, Translation, and Adaptation in a Global Ecology (ADVANTAGE)—are intentional with the goal of being impactful; our efforts also synergize with the NIH’s CCH Initiative [21,24].

Specifically, the goals of CHANGE are to: (i) effectively respond to challenges that may arise as a result of experience, new knowledge, or changing conditions in the field; and (ii) create, implement, and sustain evidence-informed, context-specific, safe, and efficacious interventions/policies/standards of care to support diet, nutrition, and health in a changing global environment. With regard to CHAIN, it focuses on the intersection of factors affecting sustainable food systems, health, and nutrition in a changing domestic and global environment. And finally, the ADVANTAGE Project is an effort to better understand the intersection of food systems, diet, nutrition, and health in a changing environment by addressing the following core questions: (i) how are the current realities of CEC affecting dietary choices/patterns and relevant aspects of the food system and what are the implications for specific public health outcomes of interest?; (ii) how can we apply an ecological approach to assessing the nature and impact of these relationships?; and (iii) how can we best translate the evidence generated to support dietary guidance to promote health and prevent disease? Furthermore, not only do our efforts align and synergize with many federal initiatives in this space (e.g., the Interagency Crosscutting Group on Climate Change and Human Health) [23], but they, importantly, are also identifying and addressing key scientific gaps and informing a research agenda to provide an evidence base to move the field forward.

## 6. Summary

Our physical surroundings are rapidly changing, and a PGNB priority is to support science addressing the interactions among health, nutrition, food systems, climate, and the environment to provide the evidence base to inform relevant stakeholders. CEC and its bidirectional relationship with food systems is indeed a critical global health challenge. But this challenge also presents an opportunity to move the science forward in a way that both preserves the planet and humankind.

## Data Availability

Not applicable.
